# Pathologic complete response and survival in HER2-low and HER2-zero early breast cancer treated with neoadjuvant chemotherapy

**DOI:** 10.1007/s12282-023-01490-1

**Published:** 2023-08-10

**Authors:** Silvia Mihaela Ilie, Nathalie Briot, Guillaume Constantin, Nicolas Roussot, Alis Ilie, Anthony Bergeron, Laurent Arnould, Françoise Beltjens, Isabelle Desmoulin, Didier Mayeur, Courèche Kaderbhai, Audrey Hennequin, Clémentine Jankowski, Marie Martine Padeano, Helène Costaz, Alix Amet, Charles Coutant, Bruno Coudert, Aurélie Bertaut, Sylvain Ladoire

**Affiliations:** 1 Department of Medical Oncology, Georges Francois Leclerc Cancer Centre, 1 Rue du Professeur Marion, 21000 Dijon, France; 2 Unit of Methodology and Biostatistics, Georges Francois Leclerc Cancer Centre, Dijon, France; 3 Platform of Transfer in Biological Oncology, Georges François Leclerc Cancer Center, Dijon, France; 4 Department of Biology and Pathology of Tumors, Georges Francois Leclerc Cancer Centre, Dijon, France; 5 Department of Surgical Oncology, Georges Francois Leclerc Cancer Centre, Dijon, France; 6 University of Burgundy-Franche Comté, 21000 Dijon, France; 7 INSERM U1231, 21000 Dijon, France

**Keywords:** Breast cancer, Neoadjuvant chemotherapy, Pathologic complete response, HER2 low, HER2 zero

## Abstract

**Background:**

Breast cancers without HER2 amplification but still expressing this membrane protein constitute a new entity called HER2-low tumors. It is important to characterize them in terms of sensitivity to treatment and prognosis.

**Patients and methods:**

To investigate chemosensitivity and long-term prognosis of HER2-low early breast cancer (eBC), compared to HER2-0 tumors, we retrospectively retrieved clinicopathological characteristics, response to treatment, and survival data from 511 patients treated for eBC with neoadjuvant chemotherapy (NAC) in a French cancer center between 2007 and 2018. Factors associated with the achievement of pathologic complete response (pCR) and survival were studied among hormone receptor positive (HR+) and negative (HR–) eBC.

**Results:**

A total of 280 HR+ (61% HER2-low), and 231 HR– (28% HER2-low) eBC were included. We found classical clinicopathological factors usually associated with chemosensitivity and prognosis, in both HR+ and HR– eBC. By uni- and multivariable analysis, HER2 status (low *vs* 0) was not independently associated with pCR, either in HR+ or HR– eBC. Relapse free (RFS) and overall survival (OS) were not significantly different between HER2-low and HER2-0 among HR+ tumors. In contrast, among HR– negative tumors, RFS and OS were slightly better in HER2-0 eBC by univariable but not by multivariable analysis.

**Conclusions:**

In eBC patients treated with NAC, taking into account HR expression subtype and other current clinicopathological features, HER2-low tumors did not appear to have different chemosensitivity or prognosis, compared to their HER2-0 counterparts.

**Supplementary Information:**

The online version contains supplementary material available at 10.1007/s12282-023-01490-1.

## Introduction

Approximately 15–20% of breast cancers are characterized by an oncogenic amplification of the ErbB2 gene, encoding the HER2 transmembrane protein [1]. This tumor subset has been known for almost 20 years as “HER2 positive” breast cancers (3+ by immunohistochemistry (IHC), or 2+ by IHC and ErbB2 amplification by fluorescent in situ hybridization (FISH)). This group of tumors presents some characteristics of clinical and biological aggressiveness, but its prognosis has been favorably transformed thanks to targeted therapies, such as monoclonal antibodies (trastuzumab and pertuzumab), or first-generation antibody–drug conjugates (ADCs) (like T-DM1) [2–4]. However, these pioneering therapies targeting HER2 have no clinical effect on the 80% of breast cancers that are devoid of HER2 amplification (formerly called “HER2 negative”), and including tumors expressing low levels of HER2 by IHC (1+ , or 2+ without gene amplification by FISH). This latter group of tumors is now defined as a "HER2-low" subgroup and constitutes about 50–60% of estrogen receptor-positive (HR+) tumors, and 30–40% of ER– tumors [5]. In contrast, the new generation of HER2-targeted ADCs, such as trastuzumab deruxtecan (T-DXd), have shown impressive response rates and benefits in terms of progression-free (PFS) and overall survival (OS), compared to conventional chemotherapy, in HER2-low tumors, thus expanding the proportion of breast cancers accessible to HER2-targeted therapy [6, 7].

The biology of these HER2-low tumors remains to be fully described, and the question arises whether they are a different clinical entity from tumors without HER2 expression (HER2-0 by IHC), both in terms of prognosis and in terms of response to conventional treatments such as chemotherapy. The significant efficacy of second-generation anti-HER2 ADCs in the metastatic setting will probably lead to their use in the near future for the treatment of early-stage breast cancer (eBC) as an adjunct to, or in replacement of conventional chemotherapy. In this context, it is important to know whether the chemosensitivity of HER2-low tumors is different from that of HER2-0 tumors.

Chemosensitivity in breast cancer can be assessed by the achievement of pathological complete response (pCR) after neoadjuvant chemotherapy (NAC) [8, 9]. pCR is associated in some breast cancer subtypes with a better prognosis, in terms of relapse-free survival and OS [8, 9].

In this study, we assessed both the prognostic and predictive value of tumor HER2-low status (compared to HER2-0) in a large series of patients treated with NAC for eBC.

## Patients and methods

### Study design and patients

We performed a retrospective analysis among operable, early breast cancer (eBC) patients addressed between 2007 and 2018 to the Georges Francois Leclerc Cancer Centre in Dijon, France, for surgery with curative intent, and in whom NAC was indicated. Pathological complete response (pCR) was defined as ypT0/is and ypN0 on surgical specimens after NAC.

**Fig. 1:**
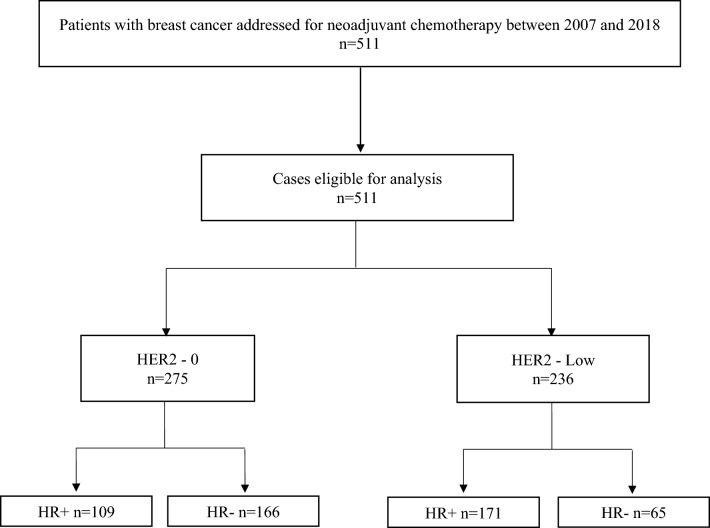
CONSORT diagram of study

Patients with HER2-amplified eBC (3+ by IHC, or 2+ with amplification by FISH) were excluded. We included adult patients (age > 18 years) with unilateral invasive, non-metastatic (stages I–III) eBC in whom the primary biopsy was evaluated by IHC for estrogen and progesterone receptors and HER2, without metachronous homo- and contralateral breast cancer, and who provided written informed consent for retrospective collection of their data (see CONSORT diagram of the study in Fig. 1). The study was conducted in accordance with the Declaration of Helsinki and approved by the Institutional Review Board and CNIL (French national commission for data privacy).

### Immunohistochemical tumor evaluation

For all the patients included in this study, the diagnosis of eBC and the immunohistochemical analyses were performed in our institution. HER2 status was determined from the pathology report derived from the analysis of diagnostic biopsies.

HER2 was assessed using standard antibodies and FISH techniques, and HER2 scoring was assessed according to the ASCO/CAP guidelines in force at the time of the patient’s recruitment [10–12]. All the stainings by immunohistochemistry were performed on Ventana/Roche autostainers (Benchmark, XT or Ultra) using A485 and then 4B5 antibodies. FISH analyses were performed using Zytovision probes. All HER2 determinations were done by a single team of experienced breast pathologists (LA, FB, AB) using a positive control for every IHC assay (i.e. positive control on every slide, and multi-tissue block with 0, 1+, 2+ and 3+ controlled tumours for every run). The team of pathologists carries out regular internal quality controls and participates in multiannual external international quality control programmes (UK NEQAS). This team of pathologists, who have regularly assessed the HER2 status of tumours in our institution, also received an excellent assessment of inter-observer reproducibility for HER2-low status determination in a recent evaluation by the French pathology quality assurance authority (AFAQUAP). Moreover, all the borderline cases in the routine diagnosis were collegially analysed to find consensus. Tumors were considered HER2-low if HER2 determination was 1+ or 2+ without HER2 amplification by FISH. Other tumors were classified HER2-0. Concerning tumor hormone receptor (HR) status, tumors were defined as HR-negative (HR–) if estrogen and progesterone receptors were expressed in < 10% of tumor cells, and HR-positive (HR+) if expressed in ≥ 10% of tumor cells.

### Statistical analysis

For clinicopathological comparisons, continuous variables are described as mean (± standard deviation, SD) or median (with minimum–maximum). Categorical variables are described as number and percentage for each modality. Percentages were calculated on complete data. Continuous variables were compared between two groups using the Student *t* test in case of normally distributed variables, and otherwise, using a Wilcoxon test. The Shapiro–Wilk test was used to check the normality of the distribution. Categorical variables were compared between groups using the Chi-square test, or Fisher’s exact test as appropriate. Multivariable analysis for pCR was performed using logistic regression, controlling for parameters with p values less than 20% in univariate analysis. The median follow-up (with range) was calculated using the reverse Kaplan–Meier method. The Kaplan–Meier method was used to estimate survival rates and median survival times and their associated 95% confidence intervals. Relapse-free survival (RFS) was defined as the time in months between the date of breast cancer diagnosis and the first recurrence of either locoregional or distant metastasis, or death. Overall survival (OS) was defined as the time in months between breast cancer diagnosis and death from any cause, or last follow-up. Survival curves were compared using the log-rank test. Cox univariate and proportional hazards multivariate regression was used to determine independent predictive factors of survival. Co-variables with a *p* value < 0.20 by univariable analysis, and HER2 status (HER2-low vs HER2-0) were included in the statistical model. Variables with more than 20% missing data were not included in the multivariate model. Backward selection method, with an exit threshold at 5% was used. Correlation between eligible variables was tested. In case 2 variables were correlated, the most significant one was kept for the multivariate model. Statistical analyses were performed using SAS version 9.4 (SAS Institute Inc., Cary, NC). Statistical tests were two-sided and the threshold of significance was fixed at 5%.

## Results

### Patients and tumors

We identified a total of 511 patients with HER2 non-amplified eBC who met the inclusion criteria, and were treated with NAC between 2007 and 2018 in our centre. Median age at diagnosis was 51.6 years ([23.6–86.3] years). In the overall population, 280 patients (54.8%) had HR+ tumors, and 231 (45.2%) HR– tumors. Concerning tumor HER2 expression, 275 patients (53.8%) had HER-0 tumors, and 236 (46.2%) had HER2-low tumors. The proportion of HER2-low and HER2-0 tumors were not the same between HR+ and HR– eBC: 171 of HR+ tumor (61%), and 65 of HR– tumors (28%) were HER2-low (CONSORT diagram, Fig. 1). Complete baseline demographics and clinicopathological characteristics for the whole population are shown in Supplemental Table 1.

When comparing HER2-low and HER2-zero tumors among HR+ and HR– tumors respectively, we found very few significant differences. Among HR+ eBC, there was no significant difference in clinicopathological characteristics, except that in HR– eBC, tumor grade and Ki67% expression were significantly higher in HER2-0 tumors (Table 1). Taking into account HR-expression eBC subtype, there was no difference between HER2-0 and HER2-low tumors concerning tumor stage at diagnosis, (except for slight differences in cT stage among HR-negative tumors, but without any difference in cAJCC stage). Treatment modalities (type of breast surgery, type of NAC, adjuvant radiotherapy) were largely similar between HER2-0 and HER2-low among HR+ and HR– eBC, albeit with more use of anthracycline + taxane-based NAC in HR– HER2-low cases, and more patients with omission of radiotherapy in HR– HER2-low cases) (Table 1).

**Table 1:** Baseline characteristics by subgroup HR+ /HR– and HER2 status

Variables	HR+ (*N* = 280)		*p* value	HR– (*N* = 231)		*p* value
	HER2-0 (*N* = 109)	HER2low (*N* = 171)		HER2-0 (*N* = 166)	HER2low (*N* = 65)	
Age, median, range	51.4 [26.4–80.1]	53.3 [23.6–84.5]	0.6025	48.7 [26.4–86.3]	52.2 [27.6–85.4]	0.1915
Menopausal status			0.8413			0.165
Premenopausal	54 (50.9%)	82 (49.7%)		89 (56.3%)	27 (45.8%)	
Postmenopausal	52 (49.1%)	83 (50.3%)		69 (43.7%)	32 (54.2%)	
Missing	3	6		8	6	
cT stage			0.649			0.0215
T0	1 (0.9%)	0 (0.0%)		0 (0.0%)	2 (3.1%)	
T1	6 (5.5%)	6 (3.5%)		16 (9.7%)	5 (7.7%)	
T2	70 (64.2%)	116 (67.8%)		113 (68.5%)	45 (69.2%)	
T3	15 (13.8%)	20 (11.7%)		23 (13.9%)	3 (4.6%)	
T4	17 (15.6%)	29 (17.0%)		13 (7.9%)	10 (15.4%)	
Missing	0	0		1	0	
cN stage			0.6427			0.4765
N0	39 (35.8%)	54 (31.6%)		87 (53.0%)	28 (43.8%)	
N1	46 (42.2%)	78 (45.6%)		47 (28.7%)	19 (29.7%)	
N2	9 (8.3%)	20 (11.7%)		9 (5.5%)	6 (9.4%)	
N3	15 (13.8%)	19 (11.1%)		21 (12.8%)	11 (17.2%)	
Missing	0	0		2	1	
Histopathological type			0.2211			0.857
Ductal	94 (86.2%)	150 (88.2%)		160 (96.4%)	62 (95.4%)	
Lobular	14 (12.8%)	14 (8.2%)		2 (1.2%)	1 (1.5%)	
Other	1 (0.9%)	6 (3.5%)		4 (2.4%)	2 (3.1%)	
Missing	0	1		160 (96.4%)	62 (95.4%)	
SBR			0.5783			0.0088
1	10 (9.3%)	23 (13.5%)		0 (0.0%)	3 (5.0%)	
2	64 (59.8%)	97 (57.1%)		40 (25.0%)	20 (33.3%)	
3	33 (30.8%)	50 (29.4%)		120 (75.0%)	37 (61.7%)	
Missing values	2	1		6	5	
Clincial tumor stage (cAJCC)			0.2741			0.4793
I	4 (3.7%)	2 (1.2%)		10 (6.1%)	2 (3.1%)	
II	63 (57.8%)	109 (63.7%)		114 (69.1%)	43 (66.2%)	
III	42 (38.5%)	60 (35.1%)		41 (24.8%)	20 (30.8%)	
Missing	0	0		1	0	
KI67, median, range	30.0 [1.0–90.0]	20.0 [5.0–90.0]	0.2645	70.0 [15.0–100.0]	40.0 [20.0–80.0]	0.0193
Type of NAC			0.2629			0.0466
Others	7 (6.4%)	7 (4.1%)		2 (1.2%)	1 (1.5%)	
Taxanes	2 (1.8%)	0 (0.0%)		1 (0.6%)	1 (1.5%)	
Anthracyclines	81 (74.3%)	129 (75.4%)		94 (56.6%)	25 (38.5%)	
Anthracyclines and taxanes	19 (17.4%)	35 (20.5%)		69 (41.6%)	38 (58.5%)	
Breast surgery			0.0248			0.345
Radical	47 (43.1%)	87 (50.9%)		51 (30.7%)	26 (40.0%)	
Conservative	56 (51.4%)	83 (48.5%)		114 (68.7%)	39 (60.0%)	
Missing	6 (5.5%)	1 (0.6%)		1 (0.6%)	0 (0.0%)	
ypT stage			0.8797			0.2403
T0	3 (2.9%)	7 (4.2%)		72 (47.4%)	20 (35.1%)	
T1	55 (53.9%)	87 (51.8%)		49 (32.2%)	27 (47.4%)	
T2	40 (39.2%)	62 (36.9%)		25 (16.4%)	8 (14.0%)	
T3	3 (2.9%)	8 (4.8%)		6 (3.9%)	2 (3.5%)	
T4	1 (1.0%)	4 (2.4%)		14	8	
Missing	7	3		72 (47.4%)	20 (35.1%)	
ypN stage			0.9228			0.0179
N0	33 (33.0%)	50 (31.6%)		122 (80.3%)	36 (64.3%)	
N1	37 (37.0%)	65 (41.1%)		21 (13.8%)	12 (21.4%)	
N2	22 (22.0%)	31 (19.6%)		8 (5.3%)	4 (7.1%)	
N3	8 (8.0%)	12 (7.6%)		1 (0.7%)	4 (7.1%)	
Missing	9	13		14	9	
pathologic tumor stage (pAJCC)			0.8146			0.2733
0	6 (5.6%)	6 (3.5%)		77 (47.2%)	22 (35.5%)	
I	23 (21.5%)	42 (24.6%)		39 (23.9%)	18 (29.0%)	
II	47 (43.9%)	74 (43.3%)		36 (22.1%)	14 (22.6%)	
III	31 (29.0%)	49 (28.7%)		11 (6.7%)	8 (12.9%)	
Missing values	2	0		3	3	
Adjuvant RT			1			0.0191
No	5 (4.8%)	7 (4.2%)		2 (1.3%)	5 (8.2%)	
Yes	100 (95.2%)	160 (95.8%)		156 (98.7%)	56 (91.8%)	
Missing	4	4		8	4	

*HR* hormone receptor, *HR+* hormone receptor positive, *HR–* hormone receptor negative, *SBR* Scarff Bloom Richardson grade, *AJCC* American Joint Committee on Cancer, *c* clinical, *p* pathological, *NAC* neoadjuvant chemotherapy, *RT* radiotherapy

### Pathologic complete response (pCR) according to HER2 status

**Fig. 2:**
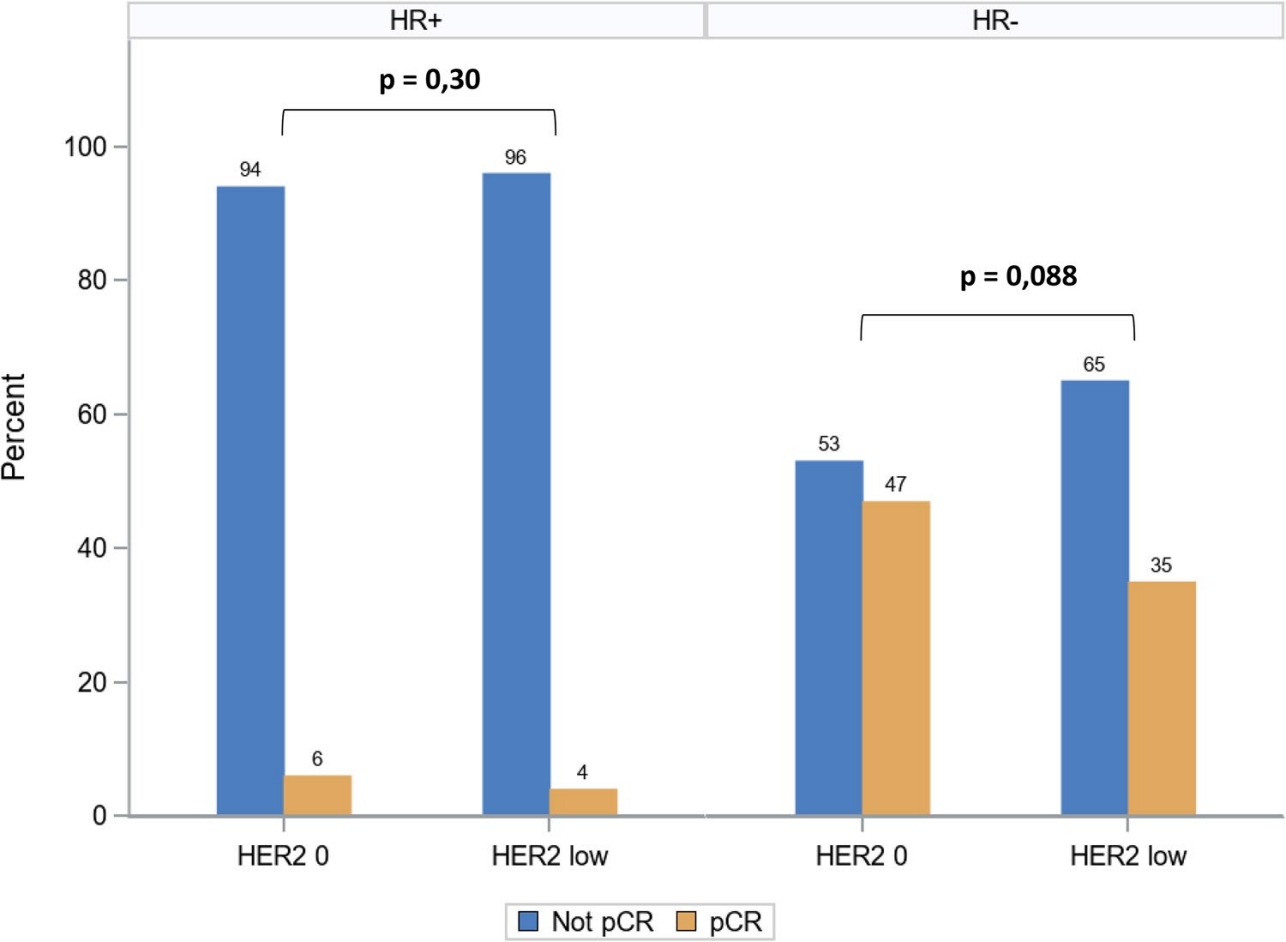
pCR rate in HR+ and in HR– breast cancer according to HER2-low and HER2-0 status

Pathologic complete response (pCR) was obtained in 111 patients (22.1%) of the overall cohort. pCR was more frequent in HR– compared with HR+ tumors (44% vs 4.3%, *p* < 0.0001). Comparing the pCR rate between HER2-0 and HER2-low tumors among HR+ and HR– tumors, there was no statistical difference in HR+ tumors (*p* = 0.30), whereas in HR– tumors, pCR tended to be more frequent in HER2-0 compared to HER2-low (47% vs 35%, *p* = 0.088) (Fig. 2). When we examined the clinicopathological factors associated with achieving pCR, we found different results among HR+ and HR– eBC: in HR+ tumors, only initial cN0 stage and higher tumor grade were associated with a higher probability of achieving pCR by univariable analysis (Table 2). By multivariable analysis, only cN0 initial stage (OR: 8.68 [1.61–46.98], *p* = 0.0121) remained associated with a higher probability of pCR (Table 2). In HR– eBC, by univariable analysis, younger age (< 50 years), cN0 initial stage, and lower cAJCC stages were associated with a higher probability of pCR, but none of these were independently associated with pCR achievement by multivariable analysis (Table 2). Of note, uni- and multivariable analyses indicated that HER2 status (low, or 0) was not associated with a significantly different probability of achieving pCR, either in HR+ , or in HR– breast cancer (Table 2).

**Table 2:** Factors associated with pCR by univariable and multivariable analyses

Variable (*n*/total)	Univariable			Multivariable		
	OR	95% CI	*p*	OR	95% CI	*p*
**Hormone receptor-positive**						
Age			0.2115			
> 50 (5/165)	1					
≤ 50 (7/113)	2.113	[0.654–6.833]				
cN			0.0023			0.0121
N + (2/185)	1			1		
N0 (10/93)	11.023	[2.363–51.427]		8.68	[1.61–46.98]	
cAJCC			0.1866			0.7878
III (1/101)	1			1		
II (11/171)	6.875	[0.874–54.066]		2.226	[0.229–21.64]	
I* (0/6)						
Grade SBR			0.0673			
1 (0/33)	1		NA			
2 (6/159)	1.749	[0.214–14.297]				
3 (6/83)	4.802	[0.598–38.551]				
HER2 status			0.4063			0.4397
HER 2 low (6/171)	1					
HER2 0 (6/107)	1.634	[0.513–5.203]		1.603	[0.485–5.304]	
**Hormone receptor-negative**						
Age			0.0267			0.0779
> 50 (41/112)	1					
≤ 50 (58/113)	1.826	[1.072–3.111]		1.661	[0.945–2.919]	
cN			0.039			0.6631
N + (40/109)	1					
N0 (58/115)	1.755	[1.029–2.995]		1.162	[0.591–2.288]	
cAJCC			0.0226			0.1822
III (19/59)	1					
II (71/154)	1.801	[0.958–3.386]		1.539	[0.705–3.359]	
I (9/12)	6.316	[1.532–26.029]		4.378	[0.902–21.24]	
Grade SBR			0.0768			0.2629
1 (1/3)	1					
2 (19/58)	0.974	[0.083–11.431]		0.667	[0.053–8.396]	
3 (77/154)	2	[0.178–22.515]		1.154	[0.095–14.01]	
HER2 status			0.114			0.3737
HER 2 low (22/62)	1					
HER2 0 (77/163)	1.628	[0.890–2.979]		1.345	[0.700–2.584]	

*OR* odds ratio, *CI* confidence interval, *AJCC* American Joint Committee on Cancer, *SBR* Scarff Bloom Richardson grade

*Not estimable: variables cannot be estimated due to small group sizes

### Relapse-free and overall survival according to HER2 status

We next examined long-term outcome of this cohort of eBC patients treated with NAC, in terms of RFS and OS, to investigate the impact of HER2 status on survival in HR+ and HR– tumors. With a median follow-up of 4.5 years (95% CI 4.1–5), the relapse rate in the overall cohort was 18.6%. Relapse rates were 21.2% and 16.4% in HER2-low and HER2-0 eBC, respectively (*p* = 0.16). Considering HR-expression subtypes, relapses were observed in 20.3% of HR+ eBC, and in 17.1% of HR– eBC (*p* = 0.35).

**Fig. 3:**
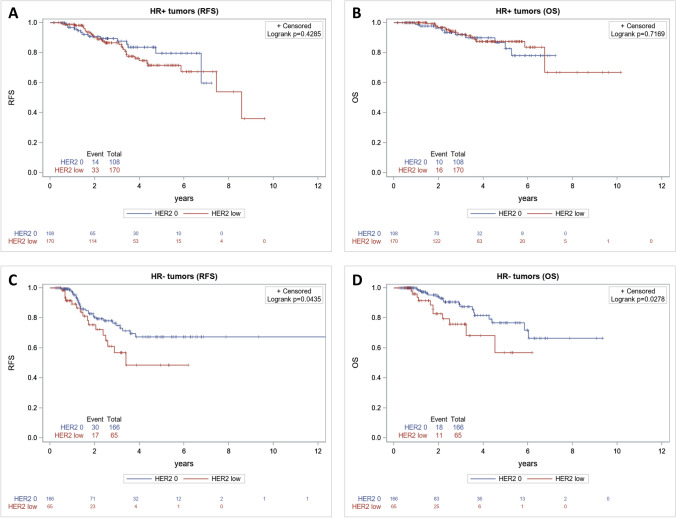
Kaplan–Meier survival curves in HR+ and HR– for RFS (**A**, **C**) and for OS (**B**, **D**)

In HR+ breast tumors, clinicopathological factors associated with worse RFS were cN + (*p* = 0.005), higher initial cAJCC stages (*p* < 0.001), and higher pAJCC stages (*p* = 0.035). Neither achievement of pCR nor HER2 status (hazard ratio (HR): 1.22; 95% CI (0.66–2.27), *p* = 0.519) was significantly associated with RFS (Table 3). Among HR+ patients, RFS curves were not statistically significantly different between HER2-0 and HER2-low tumor status (log-rank test *p* = 0.4285) (Fig. 3A). By multivariable analysis, only higher initial cAJCC stage (*p* = 0.003) was independently associated with worse RFS (Table 3). Concerning OS of patients treated for HR+ eBC, only cN+ (*p* = 0.01), and higher cAJCC stages (*p* < 0.001) were associated with OS by univariable analysis (Table 3). Here again, HER2 status was not associated with OS, and OS curves were not statistically different between HER-0 and HER2-low cases among HR+ eBC (log-rank test: *p* = 0.7169) (Table 3 and Fig. 3B). By multivariable analysis, only higher cAJCC stages remained independently associated with poorer OS (*p* < 0.001) (Table 3).

**Table 3:** Univariable and multivariable analyses for RFS and OS in HR+ and HR– breast cancer patients

Variable	Univariable RFS HR (95% CI)	*p*	Multivariable RFS HR (95% CI)	*p*	Univariable OS HR(95% CI)	*p*	Multivariable OS HR (95% CI)	*p*
**Hormone receptor-positive**								
Postmenopausal vs Premenopausal	1.2 [0.68–2.13]	0.525			1.48 [0.69–3.19]	0.318		
cN: N + vs N0	3.17 [1.42–7.09]	0.005	2.37 [0.88–6.4]	0.089	13.62 [1.85–100.55]	0.01	4.89 [0.58–40.88]	0.144
cAJCC		< 0.001		0.003		< 0.001		< 0.001
II vs I	1.07 [0.13–9.05]		0.65 [0.06–6.93]		14.55 [4.96–42.71]		10.48 [3.38–32.53]	
III vs I	4.73 [0.54–41.02]		2.56 [0.22–30.45]					
SBR	1.5 [0.82–2.77]	0.193	1.24 [0.67–2.31]	0.497	2.11 [0.97–4.61]	0.061	1.76 [0.77–3.99]	0.179
2 vs 1								
3 vs 1								
pAJCC		0.035	Not included^a^			0.152	Not included^a^	
I vs 0	0.42 [0.08–2.19]				0.51 [0.05–4.95]			
II vs 0	0.83 [0.19–3.56]				0.81 [0.10–6.34]			
III vs 0	1.56 [0.37–6.68]				1.72 [0.22–13.28]			
pCR: Yes vs No	1.08 [0.26–4.45]	0.919	3.37 [0.69–16.45]	0.133	1 [0.14–7.45]	0.996	3.46 [0.41–29.31]	0.255
HER2 low vs 0	1.22 [0.66–2.27]	0.519	1.52 [0.8–2.89]	0.207	0.8 [0.37–1.73]	0.577	1.21 [0.54–2.73]	0.649
**Hormone receptor-negative**								
Postmenopausal vs Premenopausal	2.08 [1.14–3.81]	0.017	1.8 [0.91–3.56]	0.092	3.21 [1.49–6.94]	0.003	3.09 [1.24–7.67]	0.015
N: N + vs N0	2.48 [1.28–4.79]	0.007	1.37 [0.58–3.27]	0.473	3.45 [1.31–9.08]	0.012	1.72 [0.47–6.27]	0.524
cAJCC		< 0.001		0.099		< 0.001		0.552
II vs I	0.68 [0.09–5.12]		0.34 [0.04–2.86]		0.26 [0.03–2.06]		0.24 [0.02–2.34]	
III vs I	2.47 [0.33–18.40]		0.74 [0.08–6.99]		1.15 [0.15–8.81]		0.47 [0.04–5.57]	
SBR	1.05 [0.51–2.16]	0.89	1.94 [0.85–4.44]	0.12	1.56 [0.53–4.58]	0.417	3.2 [0.9–11.29]	0.071
2 vs 1								
3 vs 1								
pAJCC		< 0.001	Not included^a^			< 0.001	Not included^a^	
I vs 0	1.99 [0.77–5.15]				1.63 [0.44–6.11]			
II vs 0	3.59 [1.52–8.48]				3.57 [1.22–10.46]			
III vs 0	21.49 [8.82–52.37]				16.97 [5.50–52.35]			
pCR Yes vs No	0.25 [0.12–0.54]	< 0.001	0.25 [0.11–0.59]	0.0014	0.25 [0.10–0.66]	0.005	0.21 [0.07–0.65]	0.007
HER2: low vs 0	1.84 [1.01–3.36]	0.047	1.42 [0.7–2.88]	0.335	2.3 [1.07–4.95]	0.032	1.28 [0.47–3.49]	0.628

*RFS* relapse-free survival, *HR* hazard ratio, *CI* confidence interval, *OS* overall survival, *AJCC* American Joint Committee on Cancer, *c* clinical, *p* pathological, *SBR* Scarff Bloom Richardson grade, *pCR* pathological complete response

^a^Not included: variables not included in multivariate models (pAJCC correlated with pCR)

In HR–breast tumors, clinicopathological factors associated with worse RFS were post-menopausal status at diagnosis (*p* = 0.017), initial cN + (*p* = 0.007), higher initial cAJCC stages (*p* < 0.001), higher pAJCC stages (*p* < 0.001), while pCR achievement was associated with better RFS (p < 0.001). HER2-low status (HR: 1.84; 95% CI (1.01–3.36), *p* = 0.047) was significantly associated with RFS (Table 3), with a significant difference between RFS curves in patients with HR– eBC (log-rank test *p* = 0.0435) (Fig. 3C). However, by multivariable analysis, only pCR achievement remained significantly and independently associated with RFS (HR: 0.25, 95% CI (0.11–0.59), *p* = 0.0014)) (Table 3). Concerning OS of patients treated for HR– eBC, post-menopausal status at diagnosis (*p* = 0.003), cN + initial stage (*p* = 0.012), higher cAJCC and pAJCC stages (*p* < 0.001), were also associated with worse OS (Table 3). Here again, pCR was associated with better OS (HR: 0.25, 95% CI (0.10–0.66), *p* = 0.005). HER2-low status was significantly associated with OS by univariable analysis (HR: 2.3, 95% CI (1.07–4.95), *p* = 0.032), as reflected by the Kaplan–Meier curves for OS (log-rank test *p* = 0.0278) (Table 3 and Fig. 3D). However, by multivariable analysis, only post-menopausal status at diagnosis (*p* = 0.015) remained significantly associated with OS, and pCR was independently associated with better OS (*p* = 0.007), with no apparent independent role for HER2 status (Table 3).

## Discussion

In this study, we show in a large series of French patients treated with NAC for localized breast cancer, that HER2-low or HER2-0 status is not independently associated either with pCR achievement, or long-term outcome in terms of relapse-free and overall survival when HR expression as well as usual clinicopathological prognostic factors are taken into account.

The nosological classification of breast cancers is currently being disrupted by the emergence of a new entity, namely “HER2-low” tumors, which is present among both HR+ and HR– tumors. For the time being, it is mainly defined by the possibility of efficient treatment with second-generation anti-HER2 ADCs such as trastuzumab deruxtecan (T-DXd) [6, 7]. Regarding the chemosensitivity of breast cancers treated with NAC, it has been shown that cancers with HER2 amplification have higher pCR rates than tumors without overexpression [13]. It is, therefore, legitimate to question whether tumors with lower levels of HER2 expression (HER2-low tumors) might have different chemosensitivity compared to HER2-0 tumors.

Initial studies seemed to show that HER2-low tumors differed from HER2-0 tumors, both as a biological entity, but also in terms of response to certain treatments such as chemotherapy, thus resulting in different prognosis [14]. In fact, with the accumulation of additional data from different patient datasets, it seems that the slight biological and prognostic differences between HER2-low and HER2-0 tumors that were initially described, are essentially related to confounding factors, due to their different respective frequencies among HR+ and HR– breast tumors [15, 16]. Indeed, as in our series, HER2-low tumors are more frequent among HR+ tumors compared with HR– tumors [5, 16]. Thus, the prevalence of HER2-low tumors seems to increase with increasing estrogen receptor expression [15]. This may explain why, in our series, the frequency of HER2 low tumors is slightly lower than in some other recent series [17]: Indeed eBC eligible for neoadjuvant strategy may have different characteristics from those of all breast tumors, including small and indolent tumors (HR expression, proliferative features…). In addition to these pathological features, molecular dissection of HER2-low and HER2-0 tumors has more recently revealed that the intrinsic transcriptomic subtypes [16], but also the common oncogenic driver mutation landscape [18] of HER2-low tumors was not significantly different from HER2-0 tumors, again when HR expression was considered. Finally, based on the current state of knowledge, the main biological differences between HER2-low and HER2-0 tumors seem to be related to HR expression rather than to the degree of HER2 expression in itself.

For this reason, in our study, we chose to perform all comparisons between HER2-low and HER2-0 tumors taking into account the HR+ or HR– subtype of breast cancer. In doing so, we found that our cohort had the expected proportion of HER2-low and HER2-0 tumors among HR+ (50–60% on average in the literature), and HR– (30–40% on average in the literature) tumors. Our series thus seems representative of the epidemiology of HER2-low and HER2-0 tumors in eBC [19]. Consistent with previously published data, in our series, HR+ tumors do not appear to have different clinicopathologic features based on HER2-0 or HER2-low status [20]. It is important to be mindful of the degree of HR expression and the threshold we used to define HR+ or HR– when interpreting these data: it has been shown that when tumors expressing HR between 1 and 9% ("HR low" tumors) were included in HR+ tumors, HER2-0 tumors tended to have higher pCR levels [15]. In our study, to avoid these biases, and to comply with the European definition of HR+ tumors, we retained only tumors expressing HR ≥ 10% in this group.

In our study, we found very low pCR rates among HR+ tumors, and without difference between HER2-0 and HER2-low tumors. For these patients, we show that the likelihood of pCR is mostly related to the clinical stage of the initial disease, in particular the existence of initial axillary lymph node invasion. Consistent with historical data in the literature, the long-term prognosis of these patients is also primarily related to the initial tumor volume (cAJCC stage and axillary lymph node involvement) [21, 22]. HER2-low or HER2-0 tumor status has no influence on the probability of pCR or on the long-term prognosis of these tumors by multivariable analysis, in line with existing data in the literature [20, 23–25]. The real value of NAC is still debated in HR+ /HER2– eBC, and the population that yields the greatest benefit from this strategy is not currently known [26]. Our findings do not support a role for HER2-low vs HER2-0 tumor status in defining a population that benefits from NAC for HR+ tumors.

Concerning HR– tumors (namely triple-negative breast cancer, TNBC), some studies have shown, like ours, a non-significant trend towards more pCR in HER2-0 tumors compared to HER2-low [15, 20, 23, 24], in contradiction with other studies [25]. Importantly, in our series, for HR– tumors, we found a significant association between HER2-0 status and a higher frequency of grade III tumors, with higher Ki67 levels. This may be a random imbalance, as previously published studies in eBC do not necessarily find such an association in HR– tumors. Conversely, these confounding factors in our series probably explain the results observed in univariable analysis for pCR (more pCR among HER2-0 tumors, because of a population enriched in proliferating tumors, and more chemosensitive), and also the survival data, with seeming better survival for HER2-0 tumors, which is logical in view of the association between pCR and RFS and OS in TNBC [8, 9]. In fact, by multivariable analyses, the impact of HER2 status on survival (RFS or OS) was no longer significant in our study after taking pCR into account. However, it should be noted that in the largest series published so far (109,588 patients), as in our series, HR+ and HR– HER2-0 eBC had statistically significantly higher pCR rates (+ 2.6% in HR+, and + 3.2% in HR–) than HER2-low tumors (a difference that persisted in multivariable analysis) [19]. Interestingly, in this work, as in the pooled analysis of 4 German randomized trials of neoadjuvant chemotherapy [14], HR– HER2-low tumors also appeared to have better survival. However, in most other large series in the literature, this effect on survival was not found [16, 20, 27–29]. These discordant results show that if a real difference exists, it is in any case minimal, without a biological substratum to explain it in the current state of knowledge. A recently published study in TNBC showed that HER2-0 tumors express the androgen receptor (AR) more frequently than HER2-low tumors [30], which may explain the trend towards poorer chemosensitivity.

In conclusion, the results of our study appear robust, as they were obtained on a large cohort of HR+ and HR– patients treated homogeneously, with tumors evaluated centrally by IHC by a single team of pathologists experienced in breast cancers. Our results add to the existing literature and show that HER2-low tumors are entities that differ little from HER2-zero tumors when the existing dichotomy between HR+ and HR– tumors is taken into account, as well as other classical clinicopathological prognostic or predictive factors of breast cancers. Taken together, these results indicate that at present, tumor HER2-low or HER2-0 status alone should not be considered when making treatment decisions in the context of neoadjuvant chemotherapy for breast cancer.

### Supplementary Information

Below is the link to the electronic supplementary material.

Supplementary file1 (DOCX 32 KB)
