# Supplementary material for: Pathologic complete response and survival in HER2-low and HER2-zero early breast cancer treated with neoadjuvant chemotherapy
Source: Breast Cancer. 2023 Aug 10;30(6):997–1007. doi: 10.1007/s12282-023-01490-1 (PMC10587331; doi:10.1007/s12282-023-01490-1)
Supplement: Supplementary file 1 — Supplementary file1 (DOCX 32 KB) [file 12282_2023_1490_MOESM1_ESM.docx]

**Supplemental Table 1: clinico-pathological characteristics of the whole cohort (N=511)**

| **Variable** | **N (%)** |
| --- | --- |
| **Age, median, range** | 51.6 [23.6 - 86.3] |
| **Menopausal status** |  |
| Premenopausal | 252 (51.6%) |
| Postmenopausal | 236 (48.4%) |
| Missing | 23 |
| **cT stage** |  |
| T0 | 3 (0.6%) |
| T1 | 33 (6.5%) |
| T2 | 344 (67.5%) |
| T3 | 61 (12.0%) |
| T4 | 69 (13.5%) |
| Missing | 1 |
| **cN stage** |  |
| N0 | 208 (40.9%) |
| N1 | 190 (37.4%) |
| N2 | 44 (8.7%) |
| N3 | 66 (13.0%) |
| Missing | 3 |
| **Histopathological type** |  |
| Ductal | 466 (91.4%) |
| Lobular | 31 (6.1%) |
| Other | 13 (2.5%) |
| Missing | 1 |
| **SBR** |  |
| 1 | 36 (7.2%) |
| 2 | 221 (44.5%) |
| 3 | 240 (48.3%) |
| Missing values | 14 |
| **Clincial tumor stage (cAJCC)** |  |
| I | 18 (3.5%) |
| II | 329 (64.5%) |
| III | 163 (32.0%) |
| Missing | 1 |
| **KI67, median, range** | 40.0 [1.0 - 100.0] |
| **Type of NAC** |  |
| Others | 17 (3.3%) |
| Taxanes only | 4 (0.8%) |
| Antracyclines + taxanes | 329 (64.4%) |
| Missing | 161 (31.5%) |
| **Breast surgery** |  |
| Radical | 211 (41.9%) |
| Conservatory | 292 (58.1%) |
| Missing | 8 |
| **ypT stage** |  |
| T0 | 102 (21.3%) |
| T1 | 218 (45.5%) |
| T2 | 135 (28.2%) |
| T3 | 19 (4.0%) |
| T4 | 5 (1.0%) |
| Missing | 32 |
| **ypN stage** |  |
| N0 | 241 (51.7%) |
| N1 | 135 (29.0%) |
| N2 | 65 (13.9%) |
| N3 | 25 (5.4%) |
| Missing | 45 |
| **pathologic tumor stage (pAJCC)** |  |
| 0 | 111 (22.1%) |
| I | 122 (24.3%) |
| II | 171 (34.0%) |
| III | 99 (19.7%) |
| Missing values | 8 |
| **Adjuvant RT** |  |
| No | 19 (3.9%) |
| Yes | 472 (96.1%) |
| Missing | 20 |
